# Successful Treatment of a Multi-Drug-Resistant Severely Pruritic Hypersensitivity Dermatitis in a Cat

**DOI:** 10.1155/2020/8897365

**Published:** 2020-10-14

**Authors:** Urszula Rzeszutek

**Affiliations:** Celia Hammond Animal Trust, 151-153 Barking Rd, Canning Town, London E16 4HQ, UK

## Abstract

A 3-year-old neutered female domestic shorthair cat was presented with a severely pruritic dermatitis. After exclusion of flea allergy dermatitis, ectoparasite infestation, retroviral infection, neoplasia, and cutaneous adverse food reaction, a diagnosis of nonflea, nonfood hypersensitivity dermatitis (NFNFHD) was made. The resolution of complicating bacterial infection and yeast overgrowth did not improve the animal's condition. Numerous antipruritic treatment modalities used during the investigation proved unsuccessful, including anti-inflammatory and immunosuppressive prednisolone doses, oclacitinib, antihistamines, ciclosporin A, and supplementation with essential fatty acids. Allergen-specific serology test results were negative. Treatment with oral dexamethasone allowed a complete resolution of clinical signs. The cat has been successfully maintained in remission for over 12 months. To the author's knowledge, this is the first case report of a cat with multi-drug-resistant NFNFHD treated successfully with dexamethasone.

## 1. Background

This report describes a challenging case of intractable severe pruritus in a cat that underwent a thorough investigation with a final diagnosis of multi-drug-resistant nonflea nonfood hypersensitivity dermatitis (NFNFHD). It highlights the potential usefulness of oral dexamethasone in cats with hypersensitivity dermatitis resistant to prednisolone and other antipruritic drugs. The author discusses the greater diabetogenic effect of dexamethasone in comparison to prednisolone and addresses the necessary monitoring of a cat receiving a long-term treatment with dexamethasone. To the author's knowledge, this is the first case report of a cat with multi-drug-resistant NFNFHD treated successfully with dexamethasone.

## 2. Case Presentation

An approximately 3-year-old neutered stray female domestic shorthair cat was presented to the Celia Hammond Animal Trust Veterinary Hospital in London with a severe dermatitis of unknown duration. Alopecia of the neck with erythema and epithelial excoriation, hair thinning in the dorsal and lateral chest area, ventral abdominal, metacarpal and metatarsal region, chin acne, and axillary and inguinal lymphadenomegaly were noted. Otherwise, clinical exam on presentation was unremarkable. No oral lesions were detected; the cat weighed 2.9 kg, had a body condition score of 4/9, and was bright, alert, and responsive. Wood's lamp examination was negative. Before the author of this report took over the case, the cat was treated with imidacloprid and moxidectin spot-on and received an injection of 0.1 mg/kg dexamethasone disodium phosphate and 8 mg/kg cefovecin sodium (Convenia; Zoetis). Oral prednisolone 1.6 mg/kg daily was prescribed.

**Figure 1:**
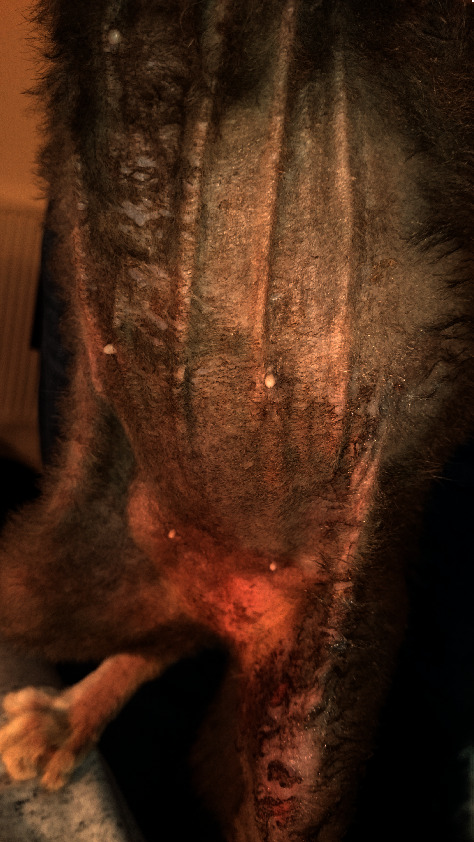
Ventral alopecia with brown adherent greasy scales on the skin of the ventral abdomen and hind limbs at the time of initial diagnostic work-up.

Observation in the hospital revealed generalized pruritus, including head and neck pruritus. Constant self-mutilation caused partial or complete alopecia and lichenification of the neck and axillary area, ventral abdomen, medial thighs, and distal hind limbs. An Elizabethan collar was used as a form of restraint to prevent self-trauma. While unable to groom in a buster collar, the cat developed brown dry crusts and adherent greasy scales on the skin of the ventral abdomen and hind limbs (Figure 1), and a brown material in the nail folds. Every time the collar was removed, the cat would lick extensively, removing all the crusts and leaving the skin erythematous and excoriated.

Upon initial lack of response to antibiotic and steroid therapy, further evaluation was undertaken. Overgrowth of yeast organisms was demonstrated by ear canal cytology and an acetate tape test taken from the skin of the ventral abdomen and axillae. No fleas or flea dirt was found on the cat's hair coat. Deep skin scrapings failed to reveal any pathogens, but due to the risk of false-negative results, an imidacloprid and moxidectin spot-on was repeated. The biochemical panel revealed no abnormalities. A complete blood count indicated mild leukocytosis with marked eosinophilia (eosinophils 6.82 109/L). A feline immunodeficiency virus and feline leukemia virus snap test (SNAP FiV/FelV Combo, IDEXX Laboratories) gave negative results. There was no evidence of a thymoma following chest radiography, and abdominal ultrasound did not detect pancreatic pathology or any other abnormalities. A fine-needle aspiration of the enlarged axillary lymph nodes was performed and sent to the Abbey Veterinary Services laboratory for evaluation. The results were consistent with hyperplasia with significant eosinophil infiltration, most likely due to allergic reaction.

To control Malassezia overgrowth, ear drops containing nystatin 100,000 I.U. (Canaural; Dechra) were applied twice daily for two weeks, and twice weekly baths with 2% miconazole/2% chlorhexidine shampoo (Malaseb; Dechra) were prescribed for three weeks. Pads containing 0.5% climbazole and 3% chlorhexidine (Douxo PYO; Ceva) were used daily on the skin lesions for three weeks. Oral supplementation of essential fatty acids (EFAs, 4 mg/cat of gamma-linoleic acid (GLA), 500 mg/cat of non-GLA omega 6 fatty acids, and 250 mg/cat of Omega 3 fatty acids) daily was prescribed. As topical treatment did not control Malassezia overgrowth, itraconazole 5 mg/kg was prescribed for 21 days.

Brown crusts and scales, assumed to be related to Malassezia overgrowth, resolved, and overall skin appearance improved, but no change in the pruritus level was observed. The buster collar had to be used at all times to prevent self-trauma. Addition of 0.5 mg/kg of oclacitinib (Apoquel; Zoetis) twice daily for 14 days and application of fluralaner (Bravecto; Merck Animal Health) did not improve the animal's condition.

At this point, the cat was rehomed by a veterinary professional working at the charity and placed in an otherwise pet-free home, where a hypoallergenic dietary trial was performed. Oral EFA supplementation was substituted with EFAs spot-on (Allerderm; Virbac). A diffuser of a synthetic analogue of the F3 fraction of feline facial pheromone (Feliway Classis; Ceva Animal Health) was installed at the apartment. During the first week of dietary trial, 10 mg/cat of diphenhydramine was given twice daily with no visible improvement and was discontinued due to bitter taste of the preparation that caused hypersalivation and stress during administration. Prednisolone was phased out during the second part of the dietary trial. No improvement was observed after 8 weeks of feeding a hydrolyzed protein diet (Royal Canin Anallergenic). No change in the pruritus level was observed after rechallenge with the cat's normal diet. Subsequent 9-week strict dietary trial with home-cooked, novel protein, two-ingredient diet (rabbit and pumpkin) proved unsuccessful. The owner reported 100% compliance during both dietary interventions.

A tentative diagnosis of nonflea, nonfood hypersensitivity dermatitis (NFNFHD) was made. Treatment with 7 mg/kg of ciclosporin A once daily (Atopica; Novartis) was prescribed. After six weeks of therapy, self-mutilation could still be managed only with a buster collar. Malassezia spp. overgrowth recurred and a distinct malodorous smell of the skin was noted.

Repeated haematology and biochemistry were performed. Further increase in eosinophils count was observed (eosinophils 7.85 109/L) with no significant change in other haematological and biochemical parameters.

**Figure 2:**
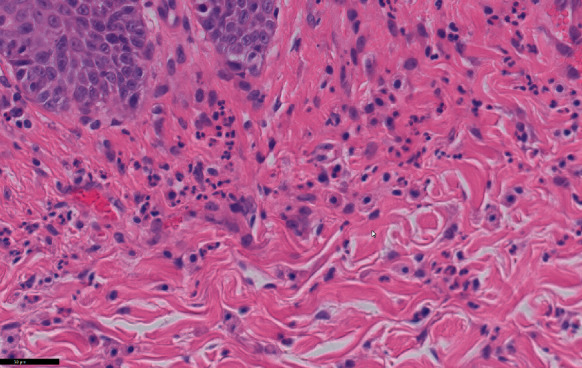
Hematoxylin and eosin photomicrograph (×40) showing eosinophils and mast cells in the dermis (courtesy of IDEXX Laboratories).

**Figure 3:**
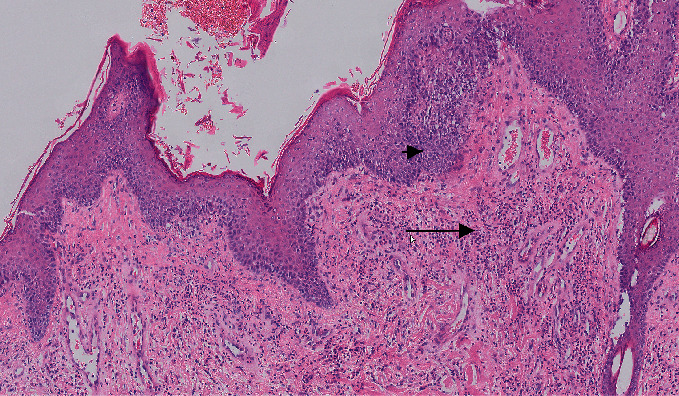
Hematoxylin and eosin photomicrograph (×10) showing epidermal hyperplasia (arrowhead) and eosinophils and mast cells in the dermis (arrow) (courtesy of IDEXX Laboratories).

At this point, punch biopsies of skin lesions were taken, with results suggesting a chronic allergic condition. Histopathology of the skin evaluated by IDEXX Laboratory revealed epithelial hyperplasia and prominent orthokeratotic and parakeratotic hyperkeratosis with multifocal crust formation, moderate dermal fibrosis, and moderate perivascular mastocytic, eosinophilic, and mildly histiocytic infiltration (Figures 2 and 3). Culture of a 1 mm punch biopsy of the skin yielded profuse growth of Malassezia spp., moderate growth of methicillin-resistant Staphylococcus aureus (MRSA), and no dermatophyte growth. Identified MRSA was resistant to cephalosporins and amoxycillin/clavulanic acid but sensitive to clindamycin with no inducible clindamycin resistance in vitro.

The cat was no longer tolerating topical therapy with 2% miconazole/2% chlorhexidine shampoo, so systemic treatment was commenced with 40 mg/kg terbinafine once daily for 4 weeks and clindamycin 11 mg/kg once daily for 3 weeks. No change in the pruritus level was noted despite the resolution of yeast and bacterial overgrowth confirmed by repeated skin culture and overall improvement of the skin appearance.

Upon resolution of infection and lack of dermatological response to different treatment modalities, immunosuppressive therapy with prednisolone (5.7 mg/kg divided in two daily doses for 5 days, followed by 2.8 mg/kg once daily for 10 days) was commenced. It failed to provide any relief in pruritus despite a decrease in eosinophil count to normal reference range, and a decrease in the size of axillary and inguinal lymph nodes. The cat still exhibited manic biting, scratching, and licking when not restraint, so the treatment was phased out.

Allergen-specific serological tests (Aller-g-detect Allergen Preliminary Panel and Malassezia IgE test, IDEXX Laboratories) proved negative for all allergens tested, i.e., mites/moulds, grass and weeds, trees, and Malassezia pachydermatis. Subsequently, an attempt to rule out the nonhistaminic origin of the intractable pruritus was made via a 3-week trial of 2 mg/kg amitriptyline and 10 mg/kg gabapentin twice daily which failed to provide any relief to the cat.

Finally, treatment with oral dexamethasone was started. An initial dose of 1.5 mg/cat (app. 0.4 mg/kg) daily provided a complete resolution of pruritus within three days from commencing. For the first time since the initial presentation, the owner was able to leave the cat without an Elizabethan collar without any risk of self-mutilation. After 7 days, the dose was tapered to 1 mg/cat (app. 0.3 mg/kg) daily for the next week, then to 0.75 mg/cat (app. 0.2 mg/kg) daily for the next three months, and finally to 0.5 mg/cat every other day.

## 3. Outcome and Follow-Up

**Figure 4:**
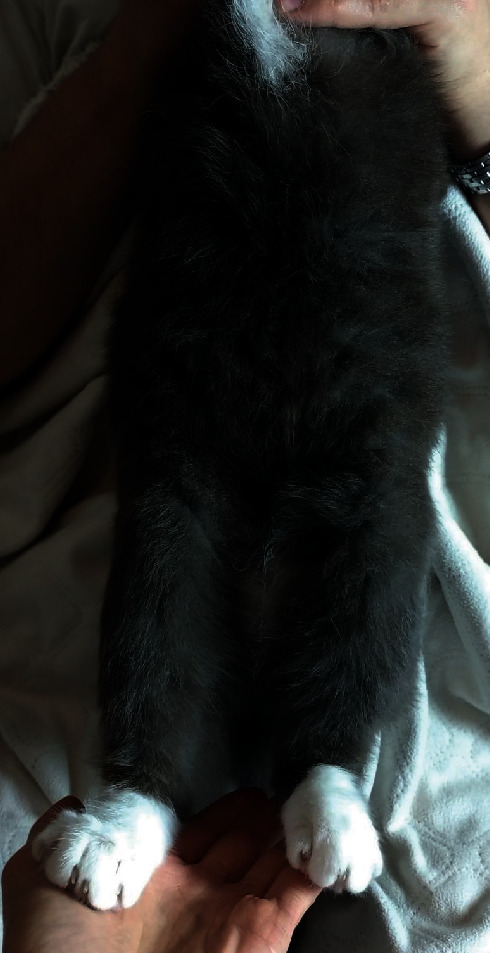
Complete hair regrowth and resolution of skin lesions on ventral abdomen and hind limbs 12 months after initiating treatment.

At the time of preparing the manuscript, the cat has been successfully maintained in remission for over 12 months (Figure 4). Polyphagia was the side effect reported by the owner, but it resolved after a dose adjustment to 0.5 mg/cat every other day. Malassezia spp. overgrowth recurred when the dose of 0.75 mg/cat was used but resolved with topical treatment with 2% miconazole/2% chlorhexidine shampoo. Every attempt to further reduce the dose resulted in a relapse of pruritus; hence, alternate-day dosing of 0.5 mg/cat is maintained.

Haematological and biochemical panel, as well as fructosamine test, was performed after six months from the start of the dexamethasone therapy, because in cats, dexamethasone may exhibit a greater diabetogenic effect than equipotent doses of prednisolone [1]. The results obtained were normal. Urine glucose checks are planned to be performed every 6 months. The frequency of monitoring may be increased depending on the clinical signs.

## 4. Discussion

The term feline nonflea, nonfood hypersensitivity dermatitis has replaced what had historically been called feline atopic dermatitis (AD) [2]. The use of the term “feline AD” remains questionable due to differences in pathogenesis and the histopathological and clinical presentation in comparison to canine and human AD [3]. As is the case with canine AD, feline NFNFHD is a diagnosis of exclusion [2, 4, 5]. Hence, parasites, infections, flea allergy dermatitis (FAD), cutaneous adverse food reactions (CAFR), and neoplastic and psychogenic causes need to be ruled out first.

In the case presented, a lack of response to flea treatment and two hypoallergenic dietary trials ruled out FAD and CAFR. Complicating bacterial infections and Malassezia spp. overgrowth were addressed with no improvement in the clinical signs. A lack of response to empirical treatment for mites and multiple negative skin scrapings rendered parasite infestation unlikely. Chest radiograph, abdominal ultrasound, and blood sample results helped to rule out paraneoplastic cutaneous syndrome, e.g., due to thymoma, pancreatic neoplasia, or biliary carcinoma [6–8]. Lymph node fine-needle aspiration and a skin biopsy were both suggestive of a chronic allergic response; however, allergen-specific serological tests were negative for the most common allergens. This highlights the equivocal role of IgE in the development of feline HD. In one study, 50% of the cats with a diagnosis of NFNFHD were negative in IgE testing against environmental allergens [9], whereas in another, 36% of clinically healthy cats had a positive immediate skin test reaction to flea extract [10].

Despite the species differences in HD pathogenesis, the treatment of feline HD is usually similar to the management of canine HD. Most of the cats with NFNFHD respond well to treatment with prednisolone or ciclosporin A [11–13] and many also to treatment with oclacitinib [13, 14]. Complete lack of response to all these three drugs (including immunosuppressive doses of prednisolone) made the case described here particularly challenging.

Manic biting, scratching, and licking exhibited by the cat, despite various antipruritic treatments, raised a suspicion of pruritus of a nonhistaminic origin. A therapeutic trial with amitriptyline and gabapentin, however, failed to improve the animal's condition.

Treatment with oral dexamethasone allowed a complete resolution of clinical signs within 3 days of commencing with a final maintenance dose of 0.12 mg/kg PO on alternate days. Every attempt to reduce the dose resulted in a relapse of pruritus. At the time of writing, the cat is responding well to the maintenance dose with no recurrence of pruritus.

To the author's knowledge, this is the first case report of a cat with multi-drug-resistant NFNFHD treated successfully with dexamethasone. The use of oral dexamethasone in cats with pruritic dermatitis that failed to respond to prednisolone has been anecdotally described in some dermatology textbooks. In the veterinary literature, there is one report of dexamethasone treatment in four cats with prednisolone-resistant non-thymoma-associated exfoliative dermatitis. However, the cases described in that study lacked clinical and histopathological features of hypersensitivity reaction [15]. Successful treatment of urticaria pigmentosa-like disease in one domestic shorthair cat with dexamethasone and cetirizine hydrochloride was also reported [16].

Studies evaluating the pharmacodynamics of dexamethasone in cats are lacking. Available data suggest that in felines, a different mechanism of this glucocorticoid may exist compared to humans. In one experimental study, administration of immunosuppressive doses of dexamethasone to healthy cats over three days did not result in changes in CD4+ and CD8+ T-cell counts but caused an increase in the number of monocytes and B-cells [17]. In two studies evaluating the in vitro effects of various immunosuppressive treatments on cytokine production by lymphocytes and lymphocyte proliferation in felines, dexamethasone alone did not suppress CD4+ and CD8+ T-cell proliferation [18] or production of interleukin-2 and interferon-gamma but suppressed the production of granulocyte-macrophage colony-stimulating factor [19]. To the author's knowledge, there are no similar reports evaluating pharmacodynamic properties of prednisolone in felines; hence, it remains unknown whether the aforementioned studies indicate a different mechanism specific to dexamethasone in felines or just a general difference in the mechanism of corticosteroids in this species compared to humans.

One study evaluating the diabetogenic effects of equipotent doses of prednisolone and dexamethasone in healthy cats showed a greater glucosuria prevalence and fructosamine concentration in dexamethasone-treated cats [1]. The animals in the study were treated with immunosuppressive doses of prednisolone and dexamethasone over 56 days without dose tapering, which is an unlikely scenario in a clinical setting. Nevertheless, urine glucose and blood fructosamine concentrations should be periodically checked in cats receiving dexamethasone for a long time. Special caution should be exercised when predisposing factors are present at the start of the treatment, e.g., obesity.

## 5. Take-Home Messages

- Oral dexamethasone can be considered a therapeutic option in cats with intractable pruritus refractory to prednisolone
- This drug should not be used as a first-line treatment due to its potential greater diabetogenic effect
- In cats receiving dexamethasone for a long time, urine glucose and blood fructosamine concentrations should be periodically checked. The frequency of glycaemia monitoring should depend on the risk factors, dosage of dexamethasone used, and clinical signs
